# No difference between CT- and MRI-based patient-specific instrumentation for total knee arthroplasty: an updated systematic review and meta-analysis

**DOI:** 10.3389/fbioe.2025.1624600

**Published:** 2025-07-23

**Authors:** Long Shao, Xiang-Dong Wu, Yuhao Liu, Xing Wang, Chunyan Li, Kun Tao, Shicheng Wang

**Affiliations:** ^1^ Joint and Sports Medicine Center, Ningbo No. 6 Hospital, Ningbo, Zhejiang, China; ^2^ Ningbo Clinical Research Center for Orthopedics, Sports Medicine & Rehabilitation, Ningbo, Zhejiang, China; ^3^ Department of Orthopaedic Surgery, Beijing Jishuitan Hospital, Capital Medical University, Fourth Clinical College of Peking University, National Center for Orthopaedics, Beijing, China; ^4^ Beijing Research Institute of Traumatology and Orthopaedics, Beijing Jishuitan Hospital, Capital Medical University, Fourth Clinical College of Peking University, National Center for Orthopaedics, Beijing, China; ^5^ Department of Spinal Surgery, Shihezi General Hospital of the Eighth Division, Shihezi, China; ^6^ Department of Clinical Laboratory, Beijing Jishuitan Hospital, Capital Medical University, Fourth Clinical College of Peking University, National Center for Orthopaedics, Beijing, China

**Keywords:** patient-specific instrumentation, total knee arthroplasty, computed tomography, magnetic resonance imaging, alignment

## Abstract

**Object:**

Our previous systematic review of either computed tomography (CT)-based or magnetic resonance imaging (MRI)-based patient-specific instrumentation (PSI) systems in total knee arthroplasty (TKA) included literature up to June 2016. However, the quickly evolving field warranted an update. Therefore, the aim of this systematic review and meta-analysis was to provide updated, evidence-based insights comparing the outcomes of CT-based versus MRI-based PSI systems in TKA.

**Methods:**

We conducted comprehensive searches of PubMed, Embase, and the Cochrane Library databases from inception to February 2025. Prospective comparative trials that compared CT-based versus MRI-based PSI systems for TKA were included. Our predefined primary outcome was the incidence of outliers in overall coronal limb alignment. Secondary outcomes encompassed the accuracy of component alignment, operation time, and clinical outcomes.

**Results:**

Nine publications reporting seven eligible trials were identified. Six trials involving a total of 407 knees were included for qualitative analysis, with five trials suitable for quantitative meta-analysis. The integrated results revealed no significant differences between CT- and MRI-based PSI systems concerning the outlier incidence of coronal overall limb alignment, the outlier incidence of coronal/sagittal alignment of the femoral/tibial component, the angular errors of coronal overall limb alignment, the angular errors of the femoral/tibial component in the coronal plane, or incidence of change of implant size of the femoral/tibial component. However, CT-based PSI systems were associated with significantly greater angular errors in coronal limb alignment (mean difference [MD]: 0.69°; 95% CI, 0.03°–1.36°; *P* = 0.04) and a prolonged operative time (MD: 5.02 min; 95% CI, 1.26min–8.79 min; *P* = 0.009) when compared to MRI-based systems. Clinical outcomes, while not amenable to meta-analysis due to clinical heterogeneity, showed no significant differences between groups during short-to mid-term follow-up.

**Conclusion:**

This finding is inconsistent with our previous study. Contrary to our previous findings, current evidence indicates no significant difference in alignment outcomes between CT-based and MRI-based PSI systems for TKA. Additionally, short-to mid-term clinical outcomes were comparable between the two imaging modalities.

**Systematic Review Registration:**

identifier CRD42022339910.

## Background

The past decade has witnessed remarkable advancements in digital orthopedic technologies, highlighted by the rise of robot-assisted total joint replacement as a breakthrough (Halm-Pozniak et al., 2023; Youssef et al., 2024; Bini et al., 2020). However, the prohibitive initial investment required for robotic equipment, combined with additional costs associated with disposable instruments and consumables, has predominantly restricted robotic-assisted procedures to high-volume medical centers. Middle- and low-volume hospitals, constrained by financial barriers and limited economic returns, consequently encounter significant challenges in adopting this advanced technology (Barbash and Glied, 2010; Ng et al., 2023; Hua and Salcedo, 2022). In this context, patient-specific instrumentation (PSI) has emerged as a compelling alternative for total knee arthroplasty (TKA), which offers customized surgical cutting guides generated from preoperative imaging data (Mattei et al., 2016). This individualized approach provides distinct advantages, including personalized implant alignment, improved surgical accuracy, and enhanced cost-effectiveness (Gong et al., 2019; Vide et al., 2017; Jaffry et al., 2014; Hickey et al., 2023). Robotic systems typically involve high initial capital investments (approximately USD 1.2 million), whereas PSI generally requires negligible capital investment but incurs per-case expenses comparable to robotic systems (e.g., USD 1520 vs. USD 1390) (Hickey et al., 2023).

Currently, PSI blocks can be fabricated based on either magnetic resonance imaging (MRI) or computed tomography (CT), both serving as foundational imaging modalities for the preoperatively design of PSI jigs (Lionberger et al., 2014). MRI typically offers superior visualization of cartilage and soft tissues but has inherent limitations in clearly defining bony anatomy. Conversely, CT excels in accurately depicting bone structures but lacks sufficient detail in visualizing cartilage and soft tissues, potentially influencing the precision of preoperative modeling and surgical planning. Despite the widespread use of these imaging techniques, uncertainty persists regarding which modality is more effective in accurately restoring the mechanical axis and ensuring precise component alignment in TKA. Although multiple prospective trials have been conducted on this issue (Fritschy and Messerli, 2011; Asada et al., 2014; Ensini et al., 2014; Pfitzner et al., 2014; Silva et al., 2016; Schotanus et al., 2016; Kang et al., 2020; Thijs et al., 2020; Theeuwen et al., 2024), their findings have been inconsistent, emphasizing the necessity for further investigation.

To address this uncertainty, we have previously conducted a systematic review and meta-analysis to compare MRI-based PSI systems with CT-based PSI systems (Wu et al., 2017). Our initial findings indicated that MRI-based PSI systems achieved superior accuracy compared to CT-based systems regarding coronal limb axis alignment in TKA (Wu et al., 2017). Given the rapid evolution of PSI technology and the continuous influx of new evidence, we have since updated our systematic review and meta-analysis to provide clinicians and researchers with the latest available data. Furthermore, this updated review aims to offer a quantitative evaluation of the effects of PSI on short-to mid-term clinical outcomes, an aspect that has not been comprehensively addressed in previous literature.

## Methods

This updated systematic review and meta-analysis was conducted in accordance with the guidelines outlined in the *Cochrane Handbook for Systematic Reviews of Interventions* (Higgins and Green, 2011) and the Preferred Reporting Items for Systematic Reviews and Meta-Analyses (PRISMA) statement (Moher et al., 2009).

### Protocol registration

The review was prospectively registered in the PROSPERO database (CRD42023393302).

### Literature search

Two independent reviewers designed and executed a comprehensive literature search across PubMed, Embase, and the Cochrane Library databases, covering all publications from inception to February 2025, without language restrictions. The search strategy integrated exploded Medical Subject Headings (MeSH) terms and relevant keywords related to TKA, PSI, CT, and MRI, along with their variants. The detailed search strategy is provided in Supplementary Material. Additionally, the ClinicalTrials.gov registry (https://clinicaltrials.gov/) was also queried to identify ongoing or unpublished clinical trials. Reference lists of eligible studies and relevant reviews were also examined to capture additional pertinent studies.

### Selection criteria

Records were managed using EndNote^®^ version 21 (Clarivate Analytics). After removing duplicates, titles and abstracts were screened independently by two reviewers, and the full texts of potentially eligible studies were subsequently assessed by both reviewers. Disagreements were resolved through discussion until a consensus was reached. Studies were included if they fulfilled the following criteria:

Population: Patients diagnosed with end-stage knee osteoarthritis scheduled for primary TKA.

Intervention: CT-based PSI for TKA.

Comparison: MRI-based PSI for TKA.

Outcomes: At least one of the predefined outcome measures.

Study Design: Prospective randomized controlled trials (RCTs) or prospective non-randomized controlled trials (non-RCTs).

### Data extraction

Data abstraction was independently performed by the same reviewers who conducted the initial study selection. The collected information included: first author, publication year, location of study, publication journal, study design, clinical setting, number of participants, characteristics of study participants, manufacturers and imaging protocols for both CT- and MRI-based PSI, types of implants used, methods for evaluating alignment accuracy, surgeon experience, dimensional accuracy, and outcome measurements. If any aspect of the study design and conduct was unclear, the corresponding authors of the study were contacted for clarification.

### Outcome measurements

The predefined primary outcome was the incidence of outliers in coronal overall limb alignment, defined as deviations exceeding 3° from the preoperative plan (applicable to both overall alignment and individual components) (Jeffery et al., 1991; Ritter et al., 1994; Fang et al., 2009; Gromov et al., 2014). This threshold was chosen because previous studies consistently associate alignment deviations greater than 3° with increased risks of adverse clinical outcomes, including implant failure, poorer functional outcomes, and higher revision rates (Jeffery et al., 1991; Ritter et al., 1994; Fang et al., 2009; Gromov et al., 2014). Secondary outcomes included the incidence of outliers in the coronal/sagittal alignment of the femoral and tibial components, angular errors in coronal overall limb alignment, angular errors of the femoral and tibial components in the coronal plane, operation time, perioperative changes in the implant sizes of the femoral and tibial components, and clinical outcomes at follow-up.

### Quality assessment

The risk of bias for each included study was independently evaluated by two reviewers using the Cochrane risk-of-bias tool (Higgins et al., 2011). The assessment covered key domains: random sequence generation, allocation concealment, blinding of participants and personnel, blinding of outcome assessment, incomplete outcome data, selective reporting, and other potential sources of bias. Studies were classified as having a low risk of bias if all domains (excluding the blinding of participants and personnel) were rated as low risk; otherwise, they were deemed to have an unclear or high risk of bias (Higgins and Green, 2011). Any discrepancies were resolved by discussion.

### Statistical analysis

We combined the studies included in this update with those included in our previous review to determine the feasibility of performing a meta-analysis. For dichotomous outcomes, risk ratios (RRs) with 95% confidence intervals (CIs) were calculated. For continuous outcomes, mean differences (MDs) with 95% CIs were computed. Statistical heterogeneity was assessed using the *I* ^*2*^ statistic; a *P*-value ≥0.05 indicated non-significant heterogeneity, while *I* ^*2*^ values > 50% were considered indicative of substantial heterogeneity (Higgins and Thompson, 2002; Higgins et al., 2003). In account of clinical heterogeneity (e.g., variability in surgeon experience with PSI), a random-effects model was applied for data pooling. All statistical tests were two-sided, with a *P*-value <0.05 considered statistically significant. Potential publication bias was examined by visually inspecting funnel plots and statistically using Egger’s test (Macaskill et al., 2001; Egger et al., 1997). All analyses were performed using Stata version 14.0 (StataCorp LP) and Review Manager version 5.4 (Nordic Cochrane Center).

## Results

### Study selection

**FIGURE 1:**
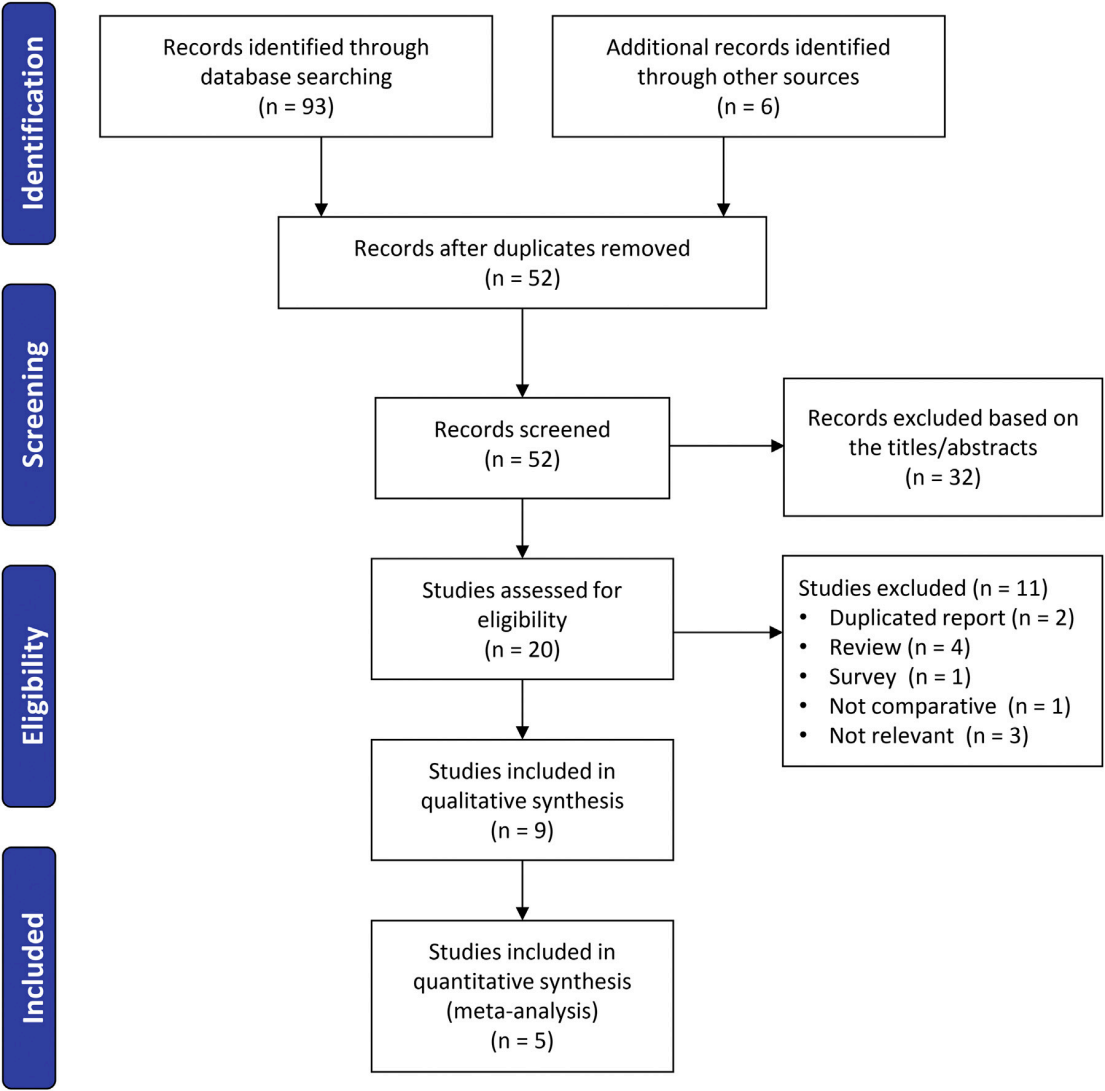
Flowchart illustrating the study selection process for the meta-analysis.

The initial literature search identified 93 records. After deduplication, 52 unique abstracts were screened. Of these, 20 full-text articles were reviewed in detail, and after applying the inclusion criteria, nine studies (Fritschy and Messerli, 2011; Asada et al., 2014; Ensini et al., 2014; Pfitzner et al., 2014; Silva et al., 2016; Schotanus et al., 2016; Kang et al., 2020; Thijs et al., 2020; Theeuwen et al., 2024) published between 2011 and 2024 were included in the systematic review. Among these, five studies (Asada et al., 2014; Ensini et al., 2014; Pfitzner et al., 2014; Schotanus et al., 2016; Kang et al., 2020) provided sufficient data for inclusion in the final meta-analysis (Figure 1).

### Description of the studies

Table 1 summarizes the main characteristics of the included trials. The nine studies represented seven distinct clinical trials conducted across seven different countries (Switzerland, Japan, Italy, Germany, Portugal, Netherlands, and South Korea). Four trials were RCTs (Ensini et al., 2014; Pfitzner et al., 2014; Schotanus et al., 2016; Theeuwen et al., 2024) while three trials were prospective non-RCTs (Fritschy and Messerli, 2011; Asada et al., 2014; Silva et al., 2016). Three trials employed both CT- and MRI-based PSI systems from the same manufacturer (Silva et al., 2016; Schotanus et al., 2016; Theeuwen et al., 2024), whereas three trials used PSI systems from different manufacturers (Asada et al., 2014; Ensini et al., 2014; Pfitzner et al., 2014), and one study did not specify the manufacturer (Fritschy and Messerli, 2011). Variations existed among manufacturers in terms of radiographic imaging protocols and prosthesis types. Furthermore, postoperative radiographic measurements used to assess lower-limb mechanical alignment accuracy and component positioning were heterogeneous, and the surgeons’ experience with conventional TKA and PSI-assisted TKA varied considerably.

**TABLE 1:** Characteristics of included prospective studies comparing CT-based versus MRI-based patient-specific instrumentation for total knee arthroplasty.

Study (country) (journal)	Study design	Clinical settings	No. of CT/MRI	Mean age, y	Male (%)	Manufacturers of CT-based PSI	Manufacturers of MRI-based PSI	Image protocol of CT-based PSI	Image protocol of MRI-based PSI	Implant of CT-based PSI	Implant of MRI-based PSI	Evaluation of accuracy	Experiences of the surgeon	Dimensional accuracy
Fritschy and Messrli (2011) ^31^ (Switzerland) (Arthroscopy)	Prospective non-randomized	NR	10	NR	NR	NR	NR	Providing data of hip, knee, ankle joints, lower limb alignment, long standing X-rays	Providing data of hip, knee, ankle joints, lower limb alignment, long standing X-rays	NR	NR	Long standing X-rays	Primary experiment was realized with 3 human specimens	No significant difference
Asada et al. (2014) ^33^ (Japan) (The Knee)	Prospective non-randomized	Knee osteoarthritis scheduled for primary TKA	20/20	75.2	17.5	Prophecy system (Wright Medical, Inc. Huntsville, AL, USA)	Visionaire system (Smith and Nephew, Inc. Memphis, TN, USA)	Icluded 1-mm high-resolution slices at the knee and selected spot images at the hip and ankle	MRI scan of the knee and a full-length anteri or/posterior radiograph	Advance Medial-Pivot Knee System (Wright Medical, Inc.Huntsville, AL, USA)	Legion Primary Knee System (Smith and Nephew, Inc. Memphis, TN, USA)	CT scanning	All surgeries were performed by the senior author	No significant differences in the accuracy
Ensini et al. (2014) ^34^ (Italy) (KSSTA)	Prospective randomized	Primary symptomatic knee osteoarthritis selected for unilateral TKA	23/22	NR	NR	Visionaire system (Smith and Nephew, Inc. London, UK)	MyKnee system (Medacta-International, Castel San Pietro, CH)	CT scan acquisitions of the hip, knee and ankle joints	MRI scan acquisitions of the knee joint and weight-bearing radiograph of the entire lower limb	GMK Total Knee System (Medacta-International, Castel San Pietro, CH)	Journey TKA prosthesis (Smith and Nephew, London, UK)	Lateral radiographs of the treated knee, a full-length standing radiograph of the lower limb in the coronal projection	Every pre-operative planning was approved by the same surgeon who performed the TKA	Both PSI systems showed good alignments, MRI performance better for a few measurements
Pfitzner et al., 2014 ^35^ (Germany) (CORR)	Prospective randomized	Primary end-stage osteoarthritis	30/30	64	43.3	TruMatch system (DePuy Orthopaedics, Inc. Warsaw, IN, USA)	Visionaire system (Smith and Nephew, Inc., Memphis, TN, USA)	CT scan of the hole leg, from hip to ankle	MRI and standing long-leg radiographs	DePuy Sigma PFC High Performance (HP) prosthesis (DePuy Orthopaedics, Inc. Warsaw, IN, USA)	Journey Bicruciate Substituting (BCS) implant (Smith and Nephew, Inc., Memphis, TN, USA)	Standing long-leg radiograph, a lateral knee radiograph, and a CT scan to measure rotation of the components	All operations were performed by a senior surgeon who had completed greater than 1000 primary TKAs and greater than 50 patient-specific instrumentation TKAs	MRI-based PSI is more accurate than CT-based PSI regarding coronal mechanical limb axis, but differences are only subtle
Silva et al. (2016) ^36^ (Portugal) (KSSTA)	Prospective non-randomized	Primary osteoarthritis, and able to undergo MRI and CT	21/23	72.1	NR	Signature system (Biomet, Inc., Warsaw, IN, USA)	Signature system (Biomet, Inc., Warsaw, IN, USA)	CT scan acquire data of the whole limb	Low-resolution images of the hip, knee and ankle,high-resolution 1-mm-thick MRI images of the knee	Vanguard Knee System (Biomet, Inc., Warsaw, IN, USA)	Vanguard Knee System (Biomet, Inc., Warsaw, IN, USA)	Underwent CT of the operated knee in the first week after the surgery to measure the components rotation	Surgery was performed with PSI by the same surgeon	No significant differences, MRI may be more accurate than CT in tibial rotation
Schotanus et al. (2018) ^37^ (Netherlands) (BJJ)	Prospective randomized	Primary end-stage osteoarthritis	70/67	68.4	36.5	Signature PSG system (Zimmer Biomet, Warsaw, IN, USA)	Signature PSG system (Zimmer Biomet, Warsaw, IN, USA)	CT scan acquire data of the whole limb	Low-resolution images of the hip, knee and ankle,high-resolution 1-mm-thick MRI images of the knee	Vanguard Cruciate Retaining TKA system (Zimmer Biomet, Bridgend, UK)	Vanguard Cruciate Retaining TKA system (Zimmer Biomet, Bridgend, UK)	Standardized long-standing weight-bearing coronal and standard sagittal digital radiograph	Four experienced knee surgeons. Each surgeon undertook at least 50 TKAs each year and had a minimum of 3 years of experience with PSG	MRI-based PSI is at least as good as CT-based PSI, MRI-based PSI is preferred for TKA
Thijs et al. (2022) (Netherlands) (KSSTA)	Prospective randomized short-term follow-up	Primary end-stage knee OA	67/57	NR	35.5	Same study as above	Same study as above	Same study as above	Same study as above	Same study as above	Same study as above	Survival rate, clinical outcome	Same study as above	No significant differences in survival rate and clinical outcome at 2-years follow-up
Theeuwen et al. (2022) (Netherlands) (EJOST)	Prospective randomized mid-term follow-up	Primary end-stage knee OA	54/44	NR		Same study as above	Same study as above	Same study as above	Same study as above	Same study as above	Same study as above	Survival rate, clinical outcome	Same study as above	No significant differences in survival rate, clinical outcome between both groups at 5-years follow-up
Kang et al. (2020) (South Korea) (Arthroplasty)	Prospective randomized	Primary knee OA only with varus deformity	35/36	38.8	8.45	Signature system (Biomet Inc., Warsaw, IN, USA)	Signature system (Biomet Inc., Warsaw, IN, USA)	1.25-mm slices of the lower extremity were obtained	Low-resolution images 5-mm spot images of the hip and ankle, high-resolution 1-mm slices of the knee	Vanguard posterior stabilized cemented TKA system (Zimmer, Biomet, Warsaw, Indiana)	Vanguard posterior stabilized cemented TKA system (Zimmer, Biomet, Warsaw, Indiana)	Standing long-leg radiograph and anterior and lateral weight-bearing radiographs of the knee	Surgery was performed by a senior surgeon	No significant differences between the MRI- and CT- based PSI systems

CT, computerized tomography; NR, not reported; MRI, magnetic resonance imaging; PSI, patient-specific instrumentation; TKA, total knee arthroplasty.

### Quality assessment

**FIGURE 2:**
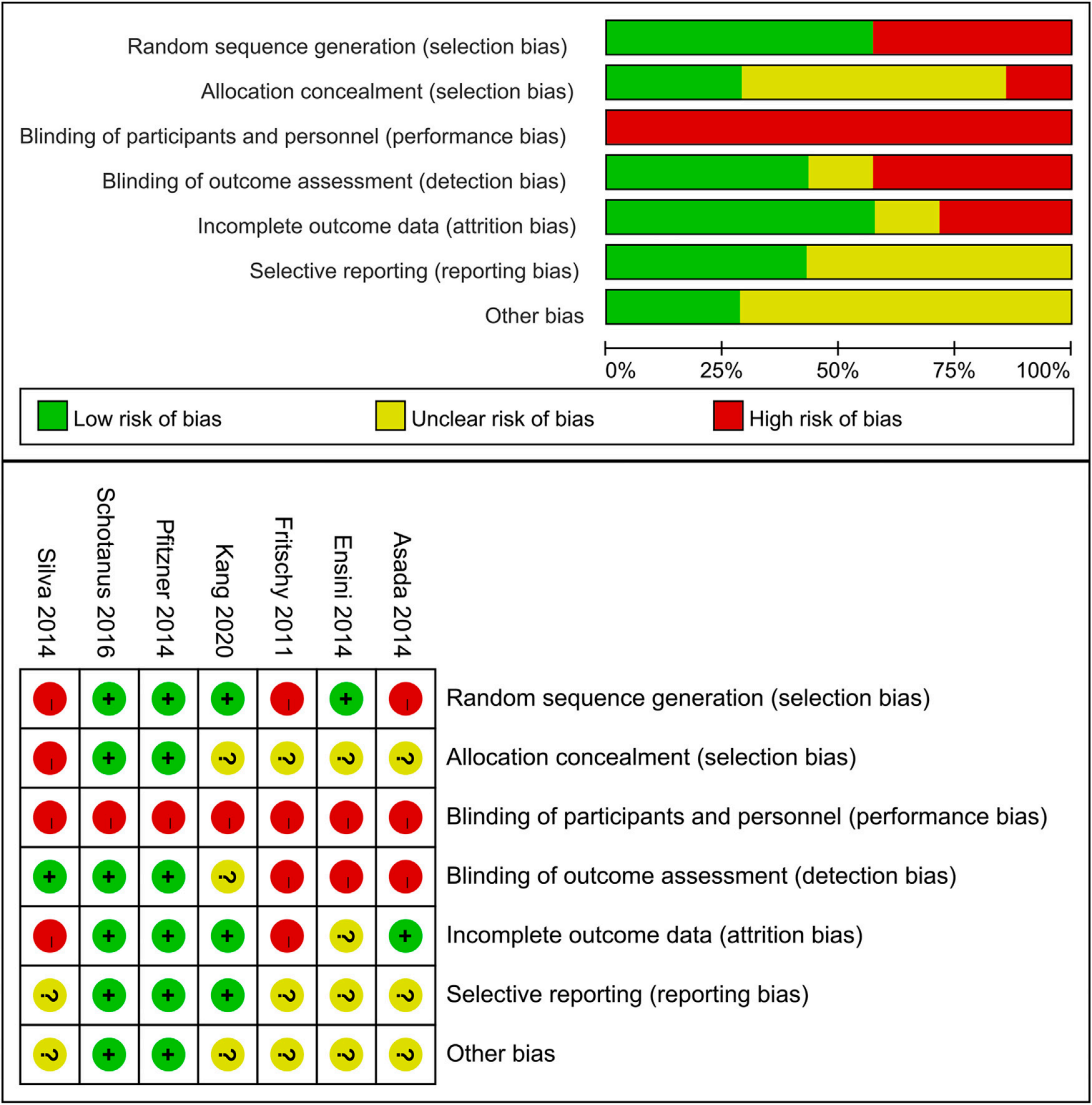
Risk of bias and Risk of bias summary across included studies.

Figure 2 summarizes the risk of bias assessment for the included studies. For the non-RCTs (Fritschy and Messerli, 2011; Asada et al., 2014; Silva et al., 2016), random sequence generation and allocation concealment were rated as unclear or high risk. Due to inherent differences between CT- and MRI-based PSI systems, especially when sourced from different manufacturers, blinding of participants was challenging. However, blinding of outcome assessment was feasible and successfully implemented in three trials (Ensini et al., 2014; Pfitzner et al., 2014; Silva et al., 2016). Overall, two trials (Pfitzner et al., 2014; Schotanus et al., 2016) were categorized as low risk of bias, whereas four (Fritschy and Messerli, 2011; Asada et al., 2014; Ensini et al., 2014; Silva et al., 2016) were considered to have a high risk of bias. Nevertheless, radiological outcomes, being relatively objective, were considered less susceptible to bias related to insufficient blinding.

### Primary outcome

**FIGURE 3:**
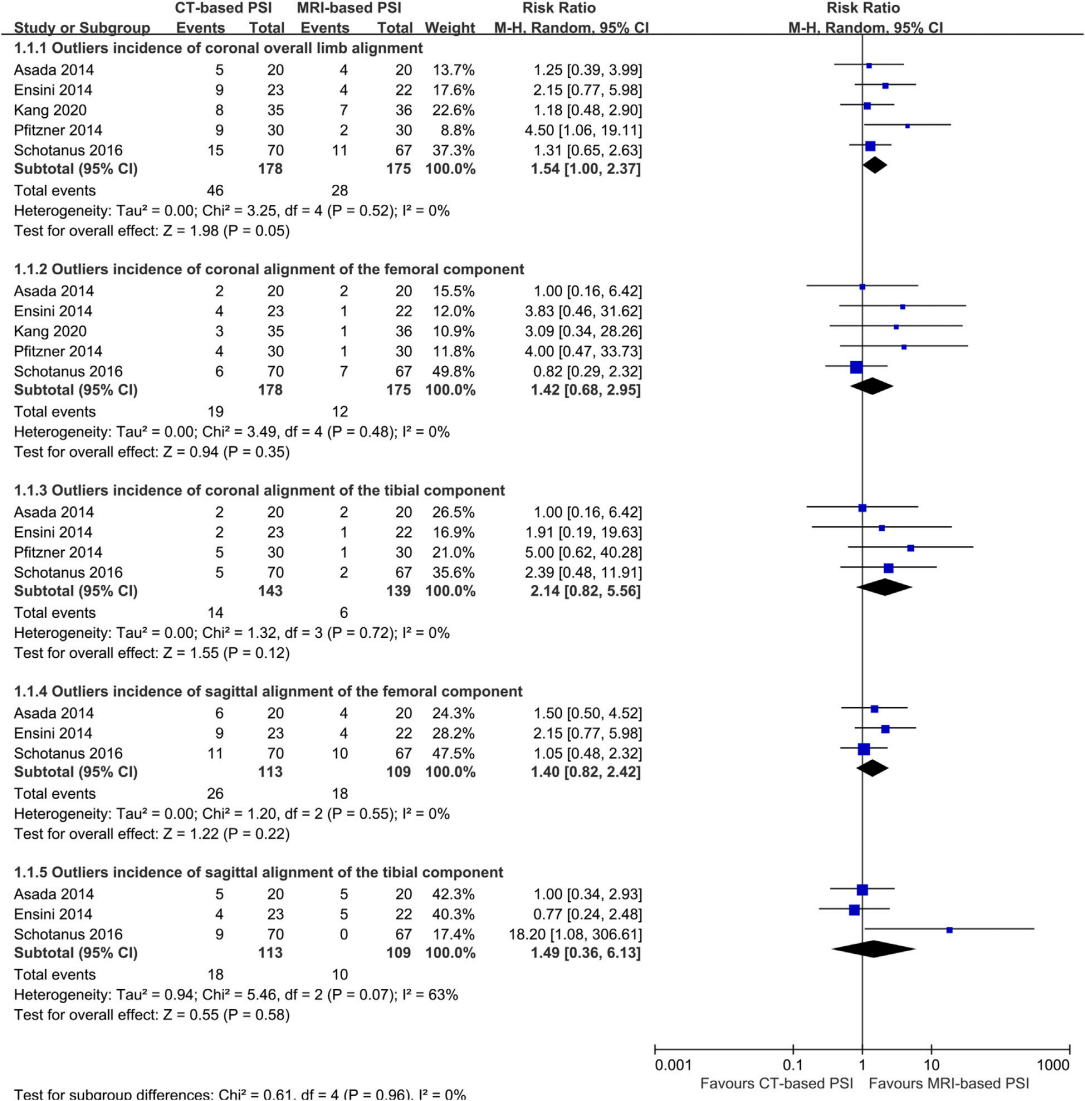
Forest plots illustrating the pooled effects comparing CT-versus MRI-based Patient-Specific Instrumentation for: outliers incidence of the coronal overall limb alignment; - outliers incidence of the coronal alignment of the femoral component; - outliers incidence of the coronal alignment of the tibial component; - outliers incidence of the sagittal alignment of the femoral component; - outliers incidence of the sagittal alignment of the tibial component; CI, confidence interval; M-H, Mantel-Haenszel.

Data on the primary outcome were available from five trials (Fritschy and Messerli, 2011; Asada et al., 2014; Ensini et al., 2014; Silva et al., 2016), encompassing a total of 353 knees. Meta-analysis indicated no significant difference in the outlier incidence of coronal overall limb alignment between MRI-based PSI and CT-based PSI was associated with a (RR: 1.54; 95% CI: 1.00–2.37; *P* = 0.055) with no heterogeneity detected (*I* ^*2*^ = 0%) (Figure 3).

### Secondary outcomes

Five trials reported the accuracy of the component alignment (Fritschy and Messerli, 2011; Asada et al., 2014; Ensini et al., 2014; Silva et al., 2016). There were no statistically significant differences between CT- and MRI-based PSI regarding the outlier incidence for:

Coronal alignment of the femoral component (RR: 1.42; 95% CI: 0.68–2.80; *P* = 0.52); coronal alignment of the tibial component (RR: 2.14; 95% CI: 0.82–5.56; *P* = 0.12); sagittal alignment of the femoral component (RR: 1.40; 95% CI: 0.82–2.42; *P* = 0.22); sagittal alignment of the tibial component (RR: 1.49; 95% CI: 0.36–6.13; *P* = 0.58); (Figure 3).

**FIGURE 4:**
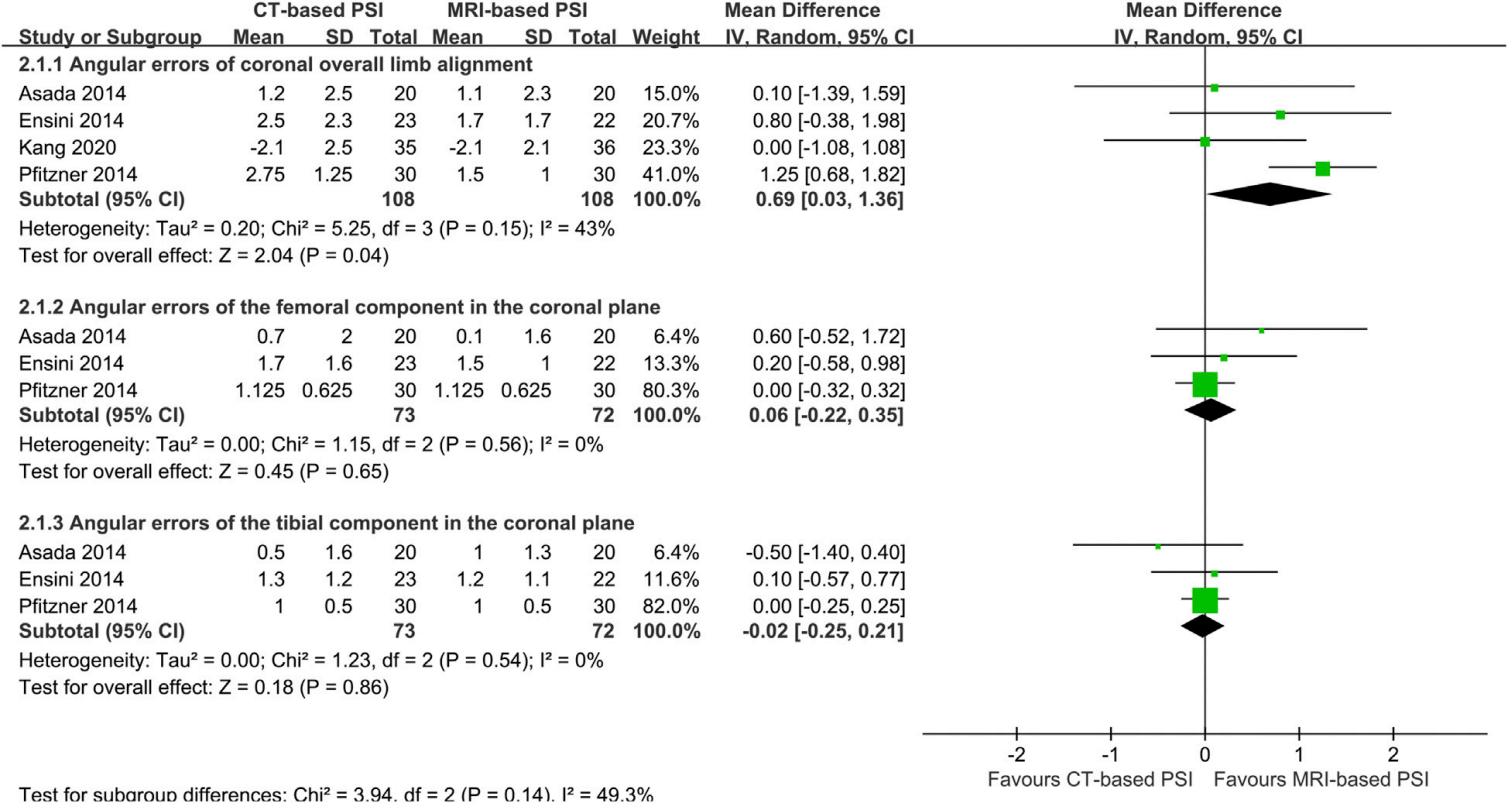
Forest plots demonstrating pooled effects comparing CT-versus MRI-based Patient-Specific Instrumentation for: - angular errors of coronal overall limb alignment; - angular errors of the femoral component in the coronal plane; - angular errors of the tibial component in the coronal plane; CI, confidence interval; M-H, Mantel-Haenszel.

**FIGURE 5:**
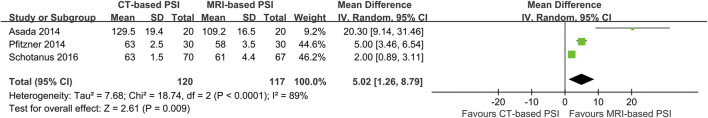
Forest plot illustrating the pooled effect of CT-versus MRI-based PSI on operation time. CI, confidence interval; I-V, Inverse Variance.

**FIGURE 6:**
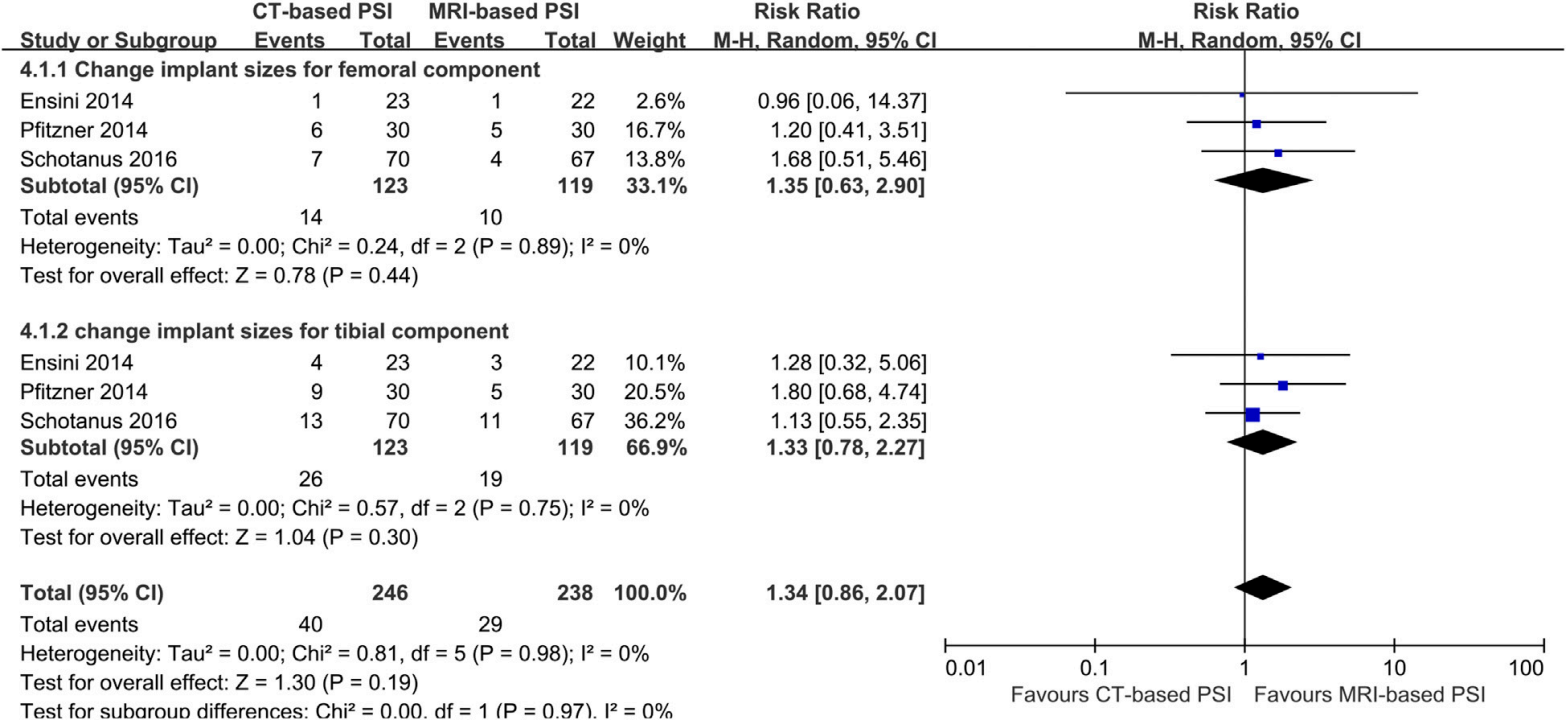
Forest plots illustrating the pooled effects comparing CT-versus MRI-based Patient-Specific Instrumentation for: - change implant size of the femoral component; - change implant size of the tibial component. CI, confidence interval; M-H, Mantel-Haenszel.

In contrast, CT-based PSI was associated with significantly greater angular errors in coronal overall limb alignment (MD: 0.69°; 95% CI: 0.0.03°–1.36°; *P* = 0.04). No significant differences were found regarding angular errors of the femoral component (MD: 0.06°; 95% CI: 0.22°–0.35°; *P* = 0.65) or the tibial component (MD: 0.02°; 95% CI: 0.25°–0.21°; *P* = 0.86) in the coronal plane (Figure 4). Furthermore, CT-based PSI was associated with a longer operation time (MD: 5.02 min; 95% CI: 1.26–8.79; *P* = 0.009) (Figure 5). No statistically significant differences were detected in the incidence of changes in implant size for either femoral (RR: 1.35; 95% CI: 0.63–2.90; *P* = 0.44) or tibial components (RR: 1.33; 95% CI: 0.78–2.27; *P* = 0.30) (Figure 6).

### Functional outcomes

Four studies provided clinical follow-up data. Pfitzner et al. reported no differences in the postoperative Knee Society pain and function scores or WOMAC scores between CT- and MRI-based PSI at 3 months postoperatively (Pfitzner et al., 2014). Similarly, Kang et al. identified no difference in Knee Society knee and functional scores, 36-item Short Form Survey scores, perioperative complications, or periprosthetic fracture rates between groups at 2 years follow-up (Kang et al., 2020). Thijs et al. observed no significant differences for the patient-reported outcome measurements (PROMs) or revision rate at 2-year follow-up (Thijs et al., 2020). Theeuwen et al. reporting mid-term follow-up on the same cohort, found a statistically significant difference only in the EuroQol-Visual Analog Scale, favoring MRI-based PSI (*P* < 0.040), while the Forgotten Joint Score and survival rates were comparable between both groups at the 5-year follow-up (Theeuwen et al., 2024).

### Publication bias

**FIGURE 7:**
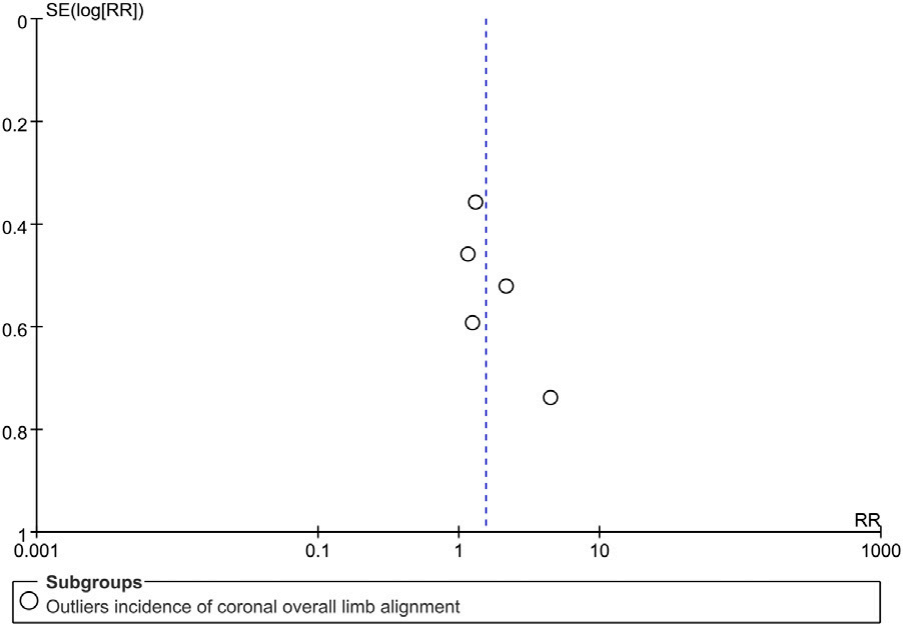
Funnel plot assessing potential publication bias for the outliers incidence of the coronal overall limb alignment. RR, risk ratio.

Visual inspection of the funnel plot for the primary outcome (Figure 7) revealed no clear evidence of publication bias, which was further supported by a non-significant Egger’s test (*P* = 0.18).

## Discussion

### Main findings

In this updated systematic review and meta-analysis, we have summarized all published evidence available between 2016 and 2025 comparing CT- and MRI-based PSI for TKA. Encouragingly, recent studies provided additional clinical follow-up data not previously available (Wu et al., 2017). Contrary to our previous review, the updated analysis suggested no significant differences between CT- and MRI-based PSI regarding either radiological or functional outcomes. Although MRI-based PSI demonstrated marginal benefits, such as a slightly lower incidence of coronal limb alignment outliers, shorter operative times, and improved EuroQol-Visual Analog Scale scores, these differences, while statistically significant, are likely clinically negligible. Specifically, a mean alignment difference of 0.69° or an operative time difference of approximately 5 min falls below commonly accepted thresholds for clinical relevance (>3° for alignment and >15 min for operative time), and thus are unlikely to meaningfully impact patient outcomes. Thus, both CT- and MRI-based PSI systems appear to provide comparable outcomes in TKA.

### Comparison with previous studies

Several previous systematic reviews and meta-analyses have compared CT‐ and MRI‐based PSI systems, yielding inconsistent results. In 2015, Stirling et al. performed a literature review and included animal studies, cadaveric studies, and clinical studies, and infer CT-based PSI might be preferable due to shorter scanning times, wider availability, and lower cost (Stirling et al., 2015). Conversely, in 2017, An et al. performed a meta-analysis of retrospective and prospective studies, and concluded that MRI-based PSI was superior regarding a lower proportion of outliers in the overall coronal limb alignment (An et al., 2017). Subsequently, our previous meta-analysis further supported the superiority of MRI-based PSI over CT-based PSI for TKA (Wu et al., 2017). In 2018, Schotanus et al. conducted an indirect comparison of CT- and MRI-based PSI for TKA, indicating that MRI-based PSI alignment was at least equivalent or potentially superior to CT-based PSI (Schotanus et al., 2018). However, in 2020, Li et al. found that CT-based PSI was preferable due to the lower rate of femoral rotational alignment outliers (Li et al., 2020).

These conflicting conclusions highlight the ongoing debate regarding the optimal imaging modality for PSI systems in TKA. Notably, our current study comprehensively integrates recent evidence and demonstrates that there were no significant differences in short-to mid-term clinical outcomes between CT-based and MRI-based PSI. Although MRI-based PSI showed statistically minor advantages, such as slightly reduced angular alignment errors and shorter operative times, the clinical significance of these findings remains limited, as these differences do not translate into meaningful variations in functional scores or revision rates. Given these uncertainties, additional long-term clinical studies are critical to clarify whether subtle differences observed between imaging modalities ultimately impact long-term implant survival and patient outcomes, providing clearer guidance for clinical practice (Klasan et al., 2020).

### Implications for clinical practice

The findings of our study bear significant implications for clinical practice. Although the results we obtained are promising, they warrant further consideration and validation to ensure their applicability and robustness in a clinical context. Based on our results, clinicians, patients, and manufacturers can confidently select either CT- or MRI-based PSI systems for TKA without significant concern for differences in radiological or functional outcomes. The comparable performance of both modalities might result from balancing advantages and limitations inherent in each imaging approach.

The comparable outcomes observed between CT- and MRI-based PSI systems may be attributed to several potential mechanisms and influencing factors. In theory, MRI provides superior visualization of cartilage and soft tissues, which can enhance the accuracy of preoperative modeling and potentially result in improved alignment outcomes. Conversely, CT provides a clearer and more detailed depiction of bone anatomy, although it inadequately visualizes cartilage. This limitation in CT imaging could lead to mismatches between the planned cutting guides and the actual resected bone surfaces, potentially affecting surgical precision. Despite these theoretical distinctions, recent comparative studies have produced inconsistent findings, possibly due to heterogeneity in study designs, varying surgeon expertise, and differences in imaging protocols and PSI manufacturers. These complexities and potential confounders highlight why previous studies, including our earlier meta-analysis, reported advantages favoring MRI-based PSI systems (Kwon et al., 2015; Koh et al., 2025), while current evidence no longer demonstrates significant clinical differences between these modalities.

However, these potential advantages of MRI-based PSI may be mitigated by factors such as the learning curve associated with PSI system design and use, as the learning curve exists for both engineers and surgeons in designing the PSI (Thi et al., 2013; Wen et al., 2022). Wen et al. proposed that engineer-based and surgeon-directed PSI designs could enhance accuracy, particularly in kinematically aligned TKA (Wen et al., 2022). Moreover, the surgeon’s experience and surgical technique can also significantly influence the clinical outcomes (Gaukel et al., 2022).

In addition, variability among manufacturers in PSI system design, including differences in imaging protocols, prosthetic designs, proprietary algorithms, and cutting-guide technologies, could introduce substantial heterogeneity, potentially masking true differences between CT- and MRI-based PSI systems. For instance, the current design philosophy of the PSI systems primarily relies on bone landmarks and rarely incorporates functional parameters adequately. However, strategies for achieving appropriate soft tissue balance, such as soft tissue releases, are typically underrepresented in PSI designs (Moerenhout et al., 2021; Deng et al., 2021). Due to these confounding factors, differences between CT- and MRI-based PSI have been diluted, resulting in minimal detectable differences in radiological and functional outcomes as demonstrated by our meta-analysis.

### Future perspectives

Innovations in TKA constantly aim to enhance surgical outcomes, improve patient functionality, and optimize cost-effectiveness. Despite the rapid global adoption of robot-assisted arthroplasty, PSI systems remain valuable. Notably, robot-assisted arthroplasty presents several drawbacks, including pin-related complications, registration errors, longer operative times, higher associated costs, and steep learning curves (Thomas et al., 2022; Fontalis et al., 2024; Pagan et al., 2024) In contrast, PSI involves the use of preoperative customized cutting guides, eliminating the necessity for intraoperative landmark registration, avoiding steps related to the intramedullary alignment of the femoral component, thus potentially reducing operation time. However, in recent years, robotic technologies have seen continuous advancements, such as the integration of sensor technologies for improved soft tissue balancing and augmented reality for intraoperative navigation (Gordon et al., 2021; Ding et al., 2024). Meanwhile, PSI has experienced relatively limited technological advancements in recent years. At present, PSI could benefit from integration with emerging technologies, such as the VERASENSE sensor, to enhance clinical outcomes in TKA (Deng et al., 2021). Further research and development efforts are necessary to explore such combinations to optimize surgical outcomes.

### Strengths and limitations

A significant strength of our meta-analysis is the inclusion of recent clinical follow-up data, providing valuable insights for clinical decision-making. Besides, we included both prospective RCTs and prospective non-RCTs clarified, which may introduce additional risk of bias but allowed a broader overview of real-world clinical practice, thus enhancing the applicability and generalizability of our findings.

However, several limitations should be acknowledged. First, the included studies utilized CT- and MRI-based PSI systems from various manufacturers, each possibly following different design philosophies, proprietary algorithms, cutting guide designs, and implant systems, thus introducing potential confounding factors and diluting observable differences between CT- and MRI-based PSI systems. Secondly, our meta-analysis is further limited by the considerable heterogeneity across included studies regarding surgeons’ experience levels and the methodologies employed for postoperative radiographic measurement, which may also affect the robustness and generalizability of our findings. Thirdly, four of the seven included trials were classified as having a high risk of bias, and inadequate blinding could further bias radiological and functional outcome assessments. Therefore, these factors necessitate a cautious interpretation of our meta-analysis results. Fourthly, the absence of long-term outcome data, particularly concerning implant survival and revision rates, necessitates a cautious interpretation of the current findings. Lastly, due to the relatively limited number of included studies and their small sample sizes, the possibility of small-study bias cannot be entirely excluded. This limitation may impact the validity and generalizability of our meta-analysis findings, warranting cautious interpretation.

## Conclusion

Our updated systematic review and meta-analysis indicate no significant differences between CT-based and MRI-based PSI systems regarding alignment accuracy or short-to mid-term clinical outcomes in TKA. This suggests that both imaging modalities are suitable for clinical utilization in TKA, although further long-term studies are required to fully validate these findings.

## Data Availability

The original contributions presented in the study are included in the article/Supplementary Material, further inquiries can be directed to the corresponding author.

## Abbreviations

CI, confidence interval; CT, computed tomography; MD, mean difference; MeSH, Medical Subject Headings; MRI, magnetic resonance imaging; PRISMA, Preferred Reporting Items for Systematic Reviews and Meta-Analyses; PROM, patient-reported outcome measurements; PSI, patient-specific instrumentation; RCT, randomized controlled trial; RR, relative risk; TKA, total knee arthroplasty.
