# Supplementary material for: No difference between CT- and MRI-based patient-specific instrumentation for total knee arthroplasty: an updated systematic review and meta-analysis
Source: Front Bioeng Biotechnol. 2025 Jul 23;13:1624600. doi: 10.3389/fbioe.2025.1624600 (PMC12325346; doi:10.3389/fbioe.2025.1624600)
Supplement: Supplementary file 1 [file DataSheet1.pdf]

Appendix 2: Database Search Strategy

	**Searched on: Aug 31th, 2024**	
**PubMed**		
**Search**	**Query**	**Results**
#1	Search "Arthroplasty, Replacement, Knee"[Mesh]	33,989
#2	Search total knee replacement[Title/Abstract]	7,182
#3	Search TKR[Title/Abstract]	2,762
#4	Search total knee arthroplasty[Title/Abstract]	28,228
#5	Search TKA[Title/Abstract]	18,313
#6	Search (((("Arthroplasty, Replacement, Knee"[Mesh]) OR total knee replacement[Title/Abstract]) OR TKR[Title/Abstract]) OR total knee arthroplasty[Title/Abstract]) OR TKA[Title/Abstract]	45,683
#7	Search patient-specific instrumentation[Title/Abstract]	545
#8	Search PSI[Title/Abstract]	22,683
#9	Search (patient-specific instrumentation[Title/Abstract]) OR PSI[Title/Abstract]	22,922
#10	Search "Tomography, X-Ray Computed"[Mesh]	509,243
#11	Search computerised tomography[Title/Abstract]	2,316
#12	Search CT[Title/Abstract]	468,066
#13	Search (("Tomography, X-Ray Computed"[Mesh]) OR computerised tomography[Title/Abstract]) OR CT[Title/Abstract]	756,981
#14	Search "Magnetic Resonance Imaging"[Mesh]	556,103
#15	Search magnetic resonance imaging[Title/Abstract]	320,679
#16	Search MRI[Title/Abstract]	349,119
#17	Search (("Magnetic Resonance Imaging"[Mesh]) OR magnetic resonance imaging[Title/Abstract]) OR MRI[Title/Abstract]	762,578
#18	Search (((((((("Arthroplasty, Replacement, Knee"[Mesh]) OR total knee replacement[Title/Abstract]) OR TKR[Title/Abstract]) OR total knee arthroplasty[Title/Abstract]) OR TKA[Title/Abstract])) AND ((patient-specific instrumentation[Title/Abstract]) OR PSI[Title/Abstract])) AND ((("Tomography, X-Ray Computed"[Mesh]) OR computerised tomography[Title/Abstract]) OR CT[Title/Abstract])) AND ((("Magnetic Resonance Imaging"[Mesh]) OR magnetic resonance imaging[Title/Abstract]) OR MRI[Title/Abstract])	40

**Cochrane**		
Search	Query	Results
#1	total knee replacement':ti,ab,kw	6,501
#2	"TKR":ti,ab,kw	868
#3	total knee arthroplasty':ti,ab,kw	8,880
#4	"TKA":ti,ab,kw	4,127

#5	#1 or #2 or #3 or #4	10,543
#6	patient-specific instrumentation':ti,ab,kw	213
#7	"PSI":ti,ab,kw	1,143
#8	#6 or #7	1,279
#9	computerised tomography':ti,ab,kw	1,346
#10	"CT":ti,ab,kw	84,764
#11	#9 or #10	85,435
#12	magnetic resonance imaging':ti,ab,kw	35,614
#13	"MRI":ti,ab,kw	36,133
#14	#12 or #13	49,783
#15	#5 and #8 and #11 and #14	21

**EMBASE**		
**Search**	**Query**	**Results**
#1	total knee replacement':ab,ti	8,780
#2	tkr:ab,ti	4,109
#3	total knee arthroplasty':ab,ti	30,392
#4	tka:ab,ti	20,179
#5	#1 OR #2 OR #3 OR #4	41,587
#6	patient-specific instrumentation':ab,ti	520
#7	psi:ab,ti	18,204
#8	#6 OR #7	18,415
#9	computerised tomography':ab,ti	3,397
#10	ct:ab,ti	786,656
#11	#9 OR #10	788,392
#12	magnetic resonance imaging':ab,ti	367,467
#13	mri:ab,ti	572,494
#14	#12 OR #13	763,560
#15	#5 AND #8 AND #11 AND #14	32
